# The Role of Adolescence in Development Paths Toward Suicide: Specificities and Shaping of Adversity Trajectories

**DOI:** 10.3389/fpsyt.2020.557131

**Published:** 2020-10-30

**Authors:** Charles-Edouard Notredame, Nadia Chawky, Guy Beauchamp, Guillaume Vaiva, Monique Séguin

**Affiliations:** ^1^Psychiatry Department, CHU Lille, Lille, France; ^2^PSY Lab, Lille Neuroscience & Cognition Centre, INSERM U1172, Lille University, Lille, France; ^3^Groupement d'Étude et de Prévention du Suicide, Saint-Benoît, France; ^4^Réseau Québécois sur le suicide, les troubles de l'humeur et les troubles associés, Douglas University Mental Health Institute, Verdun, QC, Canada; ^5^McGill Group for Suicide Studies, Douglas University Mental Health Institute, Verdun, QC, Canada; ^6^Centre National de Ressources et Résilience pour les Psychotraumatismes (Cn2r), Lille, France; ^7^Department of Psychology, Université du Québec en Outaouais, Gatineau, QC, Canada

**Keywords:** suicide, adolescence, adversity, trajectories, development

## Abstract

**Purpose:** Adolescence is a key period of transitions in the psychological, cognitive, neurobiological and relational domains, which is associated to high susceptibility to adverse life experiences. However, the way adolescent development alters life paths toward suicide remains unclear. Thereby, we aimed at testing whether and how adolescence interfered with the adversity trajectories of individuals who died by suicide.

**Methods:** In a sample of 303 individuals who died by suicide, longitudinal Burden of Adversity ratings were derived from extensive psychological autopsies and life trajectory narrative interviews conducted with informants. Piecewise Joint Latent Class Models allowed the identification of patterns of adversity trajectories and tested the introduction of breakpoints in life-paths. Classes inferred from the optimal model were compared in terms of socio-demographics, psychopathology, and rate of different adverse life events.

**Results:** The most accurate model derived 2 trajectory patterns with a breakpoint in early adolescence. In the first class (*n* = 39), the burden of adversity increased steadily from birth to death, which occurred at 23 (*SE* = 1.29). In the second class (*n* = 264), where individuals died at 43 years of age (*SE* = 0.96), the burden of adversity followed a similar trajectory during infancy but stabilized between 10 and 14 years and started to increase again at about 25. Childhood family instability, dependent events, exposure to suicide, intra-family sexual victimization and affective disorders at death were more frequent in class 1.

**Conclusions:** A bifurcation in trajectories between early and late suicides occurs during adolescence. The dynamic pattern of adversity during this period is a key issue to understand the developmental heterogeneity in suicide risk.

## Introduction

Among 15 to 29-year-olds, suicide represents the second most common cause of death and accounts for 8.5% of young people dying worldwide (1). Interestingly, suicidal behaviors generally appear, but also peak immediately after puberty (2), suggesting that adolescence may play a critical role in the development of suicidal risk.

Developmental approaches, which focus on how risk factors dynamically integrate at an individual level (3), offer a convenient framework to study how the socio-biological transformations of adolescence interferes with life-paths toward suicide (4). In a review of literature using a developmental framework, Turecki and Brent have consolidated the stress-diathesis approach of suicide, according to which the level of stress needed to precipitate suicidal behaviors depends on a specific vulnerability state called diathesis (5). The authors propose to categorize most influential risk factors based on their putative role in the sequence toward suicidal outcomes. They described distal risk factors as the early biological and environmental determinants that durably shape a person's vulnerability to suicide, developmental risk factors as the phenotypic expressions of this diathesis and proximal risk factors as the clinical conditions or triggering negative life events that contribute to precipitating suicidal behaviors.

From a developmental point of view, this stress-diathesis model builds upon a conception of suicide where individuals are seen as self-regulated organisms that adapt to a changing environment (6, 7). Because detected as threats, Adverse Life Experiences (ALE) elicit integrated neuro-biological reactions, as well as psychological and environmental changes, to maintain the balance (7). At the behavioral level, deviations from homeostasis trigger goal-oriented coping reactions in order to solve the adverse experience and/or minimize its subjective painful consequences (8). Due to a multifactorial vulnerability (5), some individuals may exhibit an increased probability of dysfunctional responses to stress—a condition known as diathesis (6). Suicide is then understood as the most extreme form of abnormal coping strategy due to stress exhausting or overwhelming regulatory mechanisms (9, 10).

Developmental models of suicidal behaviors still crucially lack proof of concept, since traditional epidemiological methods are ill-suited to the multi-deterministic and interactionist postulates of developmental psychopathology (3). In line with more dynamic alternative approaches (11, 12), our team has sought to take the understanding of the causal process toward suicide a step further by computing trajectories of Burden of Adversity (BA) (4, 13). Derived from extensive narrative material, the notion of BA integrates not only the ALE that a person has encountered in his/her life, but also the severity, interactions, repetitions and context of these ALE. Conceptually, the BA measures both the occurrence of adverse life events and their consequences in terms of maladaptive coping strategies and impact in functioning. Using longitudinal analysis, we previously identified two typical trajectories toward suicide (4). Both showed similar increasing patterns during young childhood; however, while one sharply accumulated high levels of adversity in the following age periods until death, the other was characterized by low-to-moderate BA during the whole lifetime. Although foreshadowing the effect of adolescence on the curves, the model didn't specifically test it.

However, the stress-diathesis model of suicide suggests the pivotal function of adolescence in trajectories toward suicide. During this age period, the status of risk factors changes: the diathesis is deemed stabilized and adversity mostly undergoes a precipitating influence. Implicitly, such a functional switch involves a transition in the nature of the stress-diathesis interplay, possibly reflecting the developmental transformations that adolescence implies. On the one hand, sensation-seeking, risk-taking, and appetence for novelty enable youth exploratory behaviors but also increase the probability of encountering ALE. On the other hand, puberty comes with dramatic maturation of neural and hormonal components of the allostatic system (14), thus altering the way individuals regulate stress responses and cope with adversity (8). Unfortunately, among the few longitudinal studies of adversity conducted so-far, none have specifically tested the transitional value of adolescence in life courses toward suicide.

In the present paper, we aimed at empirically deriving distinct developmental pathways toward suicide while examining whether and how such trajectories were affected by adolescence.

## Materials and Methods

### Sample and Recruitment Procedure

Participants were recruited in the provinces of Québec and New Brunswick, Canada. The sample came from four successive recruitment waves conducted between 2003 and 2015. We included all the cases of suicide registered by the provincial Chief Coroner's office during the corresponding periods. On average, 75% of identified cases were included. Reasons for non-inclusion were over-riding legal contingencies, absence of an informant, or lack of contacts between the deceased person and his or her family members.

Informants included in order of importance: parents, siblings, spouses or ex-spouses, and adult children. In some cases, two informants were interviewed, either at the same time or separately. This allowed to maximize the narrative recall by combining memories of experiences that occurred at different life periods.

The protocol received approval from the ethics review boards of the Douglas Mental Health University Institute, the Centre Hospitalier Universitaire Sainte-Justine and the Université du Québec en Outaouais. All informants signed a consent form.

### Data Collection

#### General Procedure

Skilled investigators conducted two to three in-depth interviews with each informant, focusing on their deceased relative. The interviews occurred between 6 and 18 months after the death, lasted 2–3 h on average and comprised three sections: exploration of sociodemographic characteristics and medical history, psychopathological investigation and inventory of ALE. Data collected during interviews was crossed-checked with medical, psychiatric, and psychosocial reports that were accessible from hospital files. These reports were obtained from the office of the Chief Coroner upon signed agreement of family members. Personal written documents belonging to the deceased and the informants, such as agendas and diaries, were also used if available.

#### Psychopathology

Each case was submitted to a post-mortem diagnostic assessment according to the psychological autopsy standards. The investigators administered the Structured Clinical Interview for DSM-IV for Axis I and Axis II disorders (SCID-I and SCID-II) to the informant, who was invited to respond in reference to his/her relative. In an ancillary validation study, Schneider et al. found that the inter-rater and test-retest reliably of the “by-proxy” diagnostic procedures used in psychological autopsies was excellent for most Axis I disorders (*k* > 0.84), and good to excellent for Axis II disorders (*k* > 0.65). In the same study, concordance between directly administered and informant-based SCID diagnosis reached *k* values above 0.65 for mood disorders, anxiety disorders and any axis I disorders, and strong agreements (> 97%) for personality disorders (15). Similar performances have been replicated in several psychological autopsy studies [e.g., (16–18)].

#### Inventory of Adverse Life Experiences and Estimation of Burden of Adversity

To collect all the possible ALE that the subjects had encountered, we carried out semi-structured conversational explorations inspired from life calendar narrative methods (19). The screening was conducted along a double axis of progression: (a) chronological, i.e., from birth to death, and (b) dimensional, i.e., across 9 predefined spheres of life: parent-child relationship and early ALE, affective live, procreation and/or siblings, academic or professional life, extended family, social life, losses, living conditions, and personal adversity. The retrospective recall was guided by visual timelines on which informants pinpointed memory anchors such as significant biographical elements. The length, frequency, severity, and context of each reported event were systematically collected. All the interviews were tape-recorded.

Collecting adverse life events with an informant is a standard method of psychological autopsies (20). However, only few evidence is available about the reliability of this practice. In a sample of 80 psychiatric inpatients admitted after a suicide attempt, Conner et al. found that the convergence between direct and proxy-based life event investigations was fair so substantial, with kappa ranging from 0.38 to 0.70 (21). In the present study, we sought to optimize these performances by guiding the retrospective recall. To do so, we used visual timelines on which informants pinpointed memory anchors such as significant biographical elements. We also encouraged them to use personal documents such as calendars and pictures.

Qualitative data collected from the narrative interviews were quantitatively transformed according to a human-rating procedure. After each interview, the investigators drafted synthetic clinical vignettes and summary visual calendars out of the subjects' interview, which were then submitted to a panel of independent expert raters. In a clinical decision process, the raters were asked to integrate, for 5-year periods, all the ALE that had occurred, their developmental context and the history of the individuals. This estimation took the form of a BA value ranging from 0 (low) to 5 (severe) based on standard definitions. The experts rated each trajectory independently before a consensus discussion.

### Data Analysis

After computing individual BA evolution, we used Joint Latent Class Modeling (JLCM) to derive typical adversity trajectories while accounting for the time-dependent risk of dying (22, 23). Each class was specified by its own latent growth parameters, estimated from the BA variance-covariance matrix, and the hazard function parameters, derived from the corresponding survival data set. The class membership probability was estimated over both the processes.

To assess whether adolescence represented a breaking point in the developmental trajectories to suicide, we then computed piecewise JLCM where the value of the latent growth parameters in each class was authorized to change as of a pre-determined timepoint (24). We decided to test both linear and quadratic segments of curves, as previous research showed that curvilinear shapes better account for BA trajectories than linear shapes (4).

**Figure 1:**
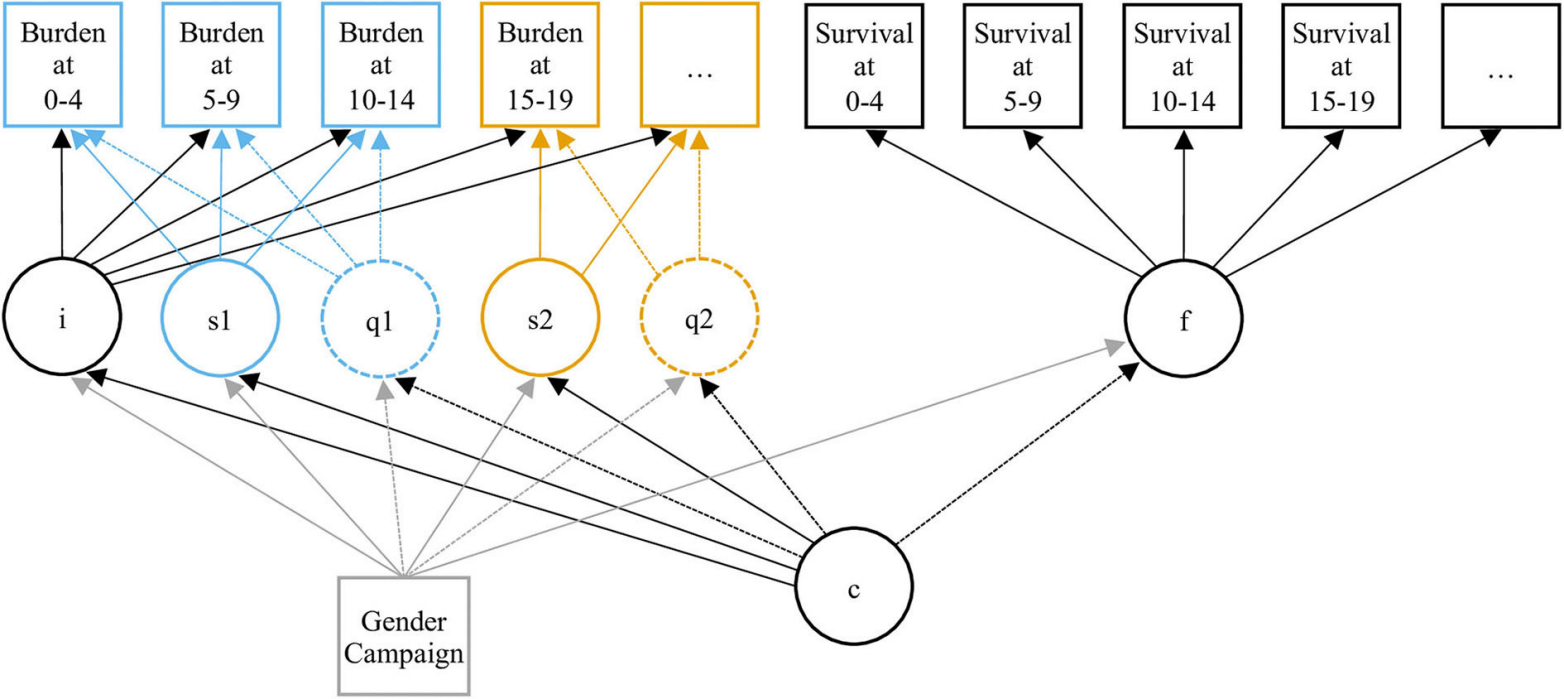
Diagram of the Joint Latent Class Model. Circles represent latent variables or processes: intercept (i), slope (q) and quadratic term (q) of the growth process, hazard function (f), and clustering variable (c). Squares represent observed variables: burden of adversity, survival data, and covariates (gender and recruitment wave). The specification of different successive slopes (s1 and s2) and quadratic terms (q1 and q2) allows piecewise Modeling. The 2 pieces of the trajectories are differentiated by their blue and yellow colors.

The resulting structure we used for our models is represented in Figure 1. We implemented all possible variations of this structure according to the following factorial development: [2, 3, or 4 classes] × [linear or linear + quadratic curve] × [no break, break at 15, 20, or 25]. A quadratic term was added only on curve segments for which the number of points was sufficient for the model to be specified. Class-specific growth and hazard parameters were adjusted on the subjects' gender and recruitment wave as time-invariant covariates.

Because the proportion of people alive progressively decreased with age, resulting in poorer information, we decided to fit our model on the 7 first time-points of available BA values, i.e., until age 34 years. Remaining non-ignorable missing data due to people dying were taken into account by the JLCM. Because JLCM account for the evolution of the outcome and for the dropouts at the same time, they allow for compensating attrition biases (25).

The detailed selection process for the best fitting model is described in Supplementary Material. Parameter estimates adjusted on time-invariant covariates corresponding to this model are available in the Supplementary Table 1. We then conducted pairwise intra- and inter-class comparisons of the growth parameter segments using Wald tests. We also compared the two classes in terms of sociodemographic characteristics, psychopathology and occurrence of ALE during distal (0–9 years old), proximal (year prior to death), and trajectory break-point periods. We used 2-sided Wilcoxon or Student tests to compare continuous distributions and Chi-square of Fisher tests to compare proportions. The alpha risk was fixed at 0.05.

Statistics were conducted with Mplus Version 7.4 (26) and R version 3.6.1 (27).

## Results

Our sample consisted of 303 individuals. Seventy percent were men. Mean age at death was 40.5 (*SD* = 16.3).

### Model Predictions

The model we retained was a 2-class, quadratic piecewise LCGM with a break at age 10–14. The fit indices were as follows: AIC = 4822.4, BIC = 4993.3, entropy = 0.91.

**Figure 2:**
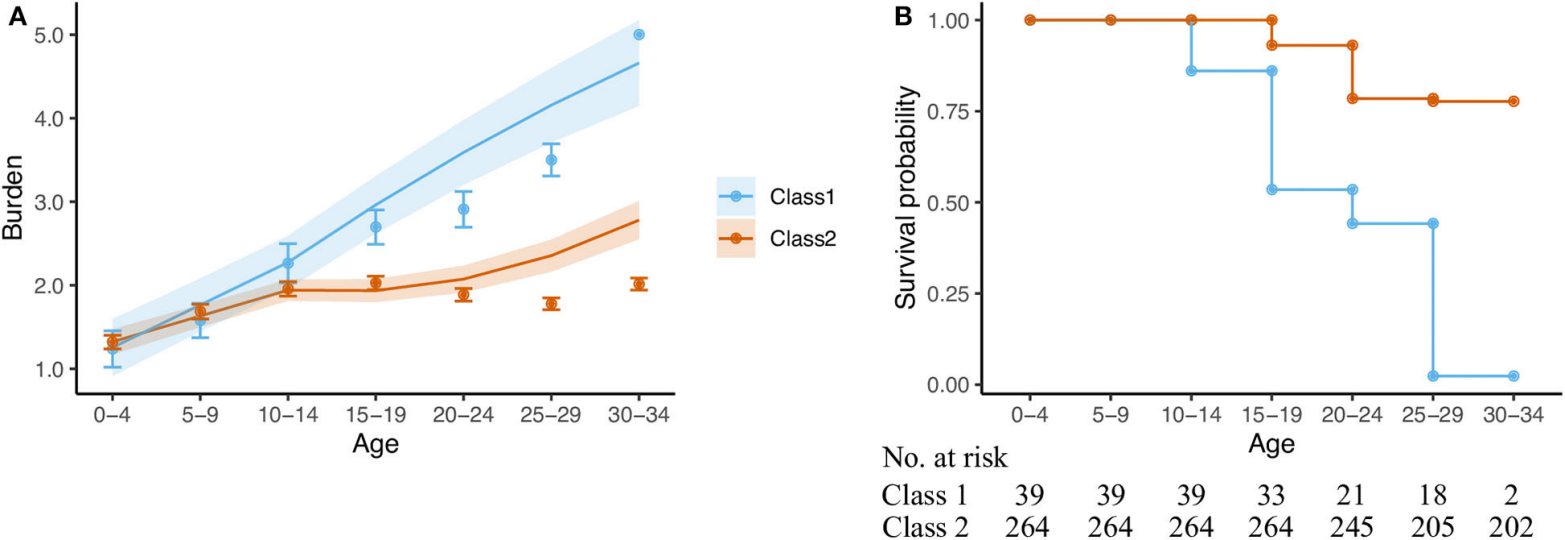
Model predictions and observed data according to the JLCM. **(A)** Trajectories of burden of adversity (mean and standard error). **(B)** Survival probability.

The predicted BA trajectories corresponding to each class are represented in Figure 2A. In the first class, which included 39 individuals (13% of the sample) who died at mean age 23.2 (*SD* = 8.0), the BA started from a non-null value of 1.25 (*SE* = 0.07) at birth and steadily increased at a rate of 0.51 (*SE* = 0.09) to 0.72 units (*SE* = 0.21) per 5 years until death. The growth was rather smooth and linear, as the quadratic term was non-significant (*p* = 0.545) and the Wald test did not reveal any significant difference between the slope parameters of the curve segments (*W* = 0.83, *p* = 0.361). The remaining 264 (87%) individuals of the sample followed a BA trajectory that also increased from birth to young adolescence at a rate of 0.31 (*SE* = 0.03) units per 5 years. However, the growth in BA significantly dampened at age 10–14 before progressively increasing again, as suggested by a non-significant slope of 0.08 (*SE* = 0.06, comparison with the slope of the first segment: *W* = 32.69, *p* < 0.001), but a significant quadratic term of 0.07 (*SE* = 0.02). In this class, death occurred at mean age 43.1 (*SD* = 15.6). Slopes significantly differed between the two classes. Size effects were modest for the first segment (*W* = 5.28, *p* = 0.022) and important for the second segment (*W* = 14.46, *p* < 0.001).

As illustrated by the survival curves in Figure 2B, the model predicted that nearly half of individuals in class 1 died before the age of 19, and almost none survived beyond 29 years of age. By contrast, more than 75% of individuals in class 2 were still alive at 30–34 years of age.

### Class Comparison

#### Sociodemographic and Psychopathological Characteristics

The sociodemographic and psychopathological characteristics of the 2 classes are presented in Table 1. As expected from the between-class discrepancy in mean age at death, we found significant differences in terms of academic level, civil status, mean number of children, and household. By contrast, the classes were comparable with respect to the gender ratio and the recruitment wave.

**Table 1:** Inter-class comparison of sociodemographic characteristics.

**Variables**	**NA *n* (%)**	**Modalities**	**Class 1 (*n* = 39)**	**Class 2 (*n* = 264)**	***p*-value^**a**^**
Age *M* (*SE*)	0 (0)		23.2 (1.29)	43.1 (0.96)	<0.001
Recruitment wave *n* (%)	0 (0)	Wave 1	14 (36)	65 (25)	0.506
		Wave 2	8 (21)	62 (23)	
		Wave 3	8 (21)	59 (22)	
		Wave 4	9 (23)	78 (30)	
Gender *n* (%)	0 (0)	Female	7 (18)	83 (31)	0.125
Civil status *n* (%)	2 (0)	Single	26 (67)	93 (35)	<0.001
		Common law	6 (15)	9 (3)	
		Married	3 (08)	88 (34)	
		Separated	4 (1)	28 (11)	
		Divorced	0 (0)	37 (14)	
		Widowed	0 (0)	7 (3)	
N of children *M* (*SE*)	20 (1)		0.4 (14)	1.2 (1)	0.001
Academic level *n* (%)	19 (1)	Post-graduate	1 (3)	9 (4)	0.002
		Graduate	0 (0)	23 (9)	
		Undergraduate	13 (36)	126 (51)	
		High school	13 (36)	40 (16)	
		Middle school	9 (25)	33 (13)	
		Elementary	0 (0)	17 (7)	
Employment *n* (%)	12 (0)	Employed	18 (51)	123 (48)	0.845
Household *n* (%)	11 (0)	Single	10 (26)	92 (36)	0.003
		With parents	9 (24)	54 (18)	
		With partner	1 (3)	43 (17)	
		With children	1 (3)	6 (2)	
		With family	3 (8)	9 (4)	
		With roommate	9 (24)	13 (5)	
		Other	5 (13)	37 (15)	

*M, Mean; NA, Not Acknowledged; SE, Standard Error*.

a *Means are compared with non-paired Wilcoxon tests. Proportions are compared with Chi-square or Fisher exact tests depending on the marginal probabilities*.

In terms of psychiatric diagnosis (see Table 2), the two classes differed only in the proportion of individuals suffering from affective disorders at death, which was significantly higher in class 2 than in class 1 (57 vs. 38%, *p* = 0.048).

**Table 2:** Inter-class comparison of psychopathological characteristics.

	**Diagnosis at death**			**Life diagnosis**		
	**Class 1 *n* (%)**	**Class 2 *n* (%)**	***p*-value^**a**^**	**Class 1 *n* (%)**	**Class 2 *n* (%)**	***p*-value^**a**^**
Bipolar disorder	0 (0)	11 (4)	0.370	0 (0)	10 (4)	0.371
Characterized depressive disorder	12 (31)	113 (43)	0.211	4 (10)	67 (25)	0.060
Any mood disorder^b^	15 (38)	150 (57)	0.048	9 (23)	103 (39)	0.081
Psychosis	4 (10)	13 (5)	0.251	3 (8)	13 (5)	0.443
Alcohol use disorder	11 (28)	63 (24)	0.697	11 (28)	80 (3)	0.937
Drug use disorder	11 (28)	44 (17)	0.128	12 (31)	49 (19)	0.119
Anxiety disorder	3 (8)	19 (7)	1.000	3 (8)	23 (9)	1
Post-traumatic stress disorder	1 (3)	7 (3)	1.000	1 (3)	10 (4)	1
Eating disorder	2 (5)	2 (1)	0.082	2 (5)	2 (1)	0.082
Attention deficit—hyperactivity disorder	0 (0)	1 (0)	1.000	0 (0)	15 (6)	0.232
Borderline personality disorder	6 (15)	36 (14)	0.963			
Any personality disorder	15 (38)	130 (49)	0.277			

*ADHD, Attention Deficit-Hyperactivity Disorder; BPD, Borderline Personality Disorder; DSM, Diagnosis Statistical Manual (as assessed by the Structured Clinical Interview for the DSM); PTSD, Post-Traumatic Stress Disorder*.

a *Proportions are compared with Chi-square or Fisher exact tests depending on the marginal probabilities*.

b *Mood disorders encompass depressive disorders, bipolar disorders, and dysthymia*.

#### Distal ALE

As observable in Table 3, left columns, the classes differed significantly in the proportion of individuals concerned by conflicts or tensions with close family members (54% in class 1 vs. 31% in class 2, *p* = 0.004), arrival of a new partner in one of the parents' lives (10% in class 1 vs. 2% in class 2, *p* = 0.018), learning disabilities (33% in class 1 vs. 18% in class 2, *p* = 0.018) and exposure to the suicide of a friend or family member (21% in class 1 vs. 7% in class 2, *p* = 0.013) between ages 0 and 9 years.

**Table 3:** Between-class comparison of the frequency of adverse events experienced in childhood (0–9), adolescence (10–14), and in the year prior to death.

	**0–9 years old**			**10–14 years old**			**Year prior death**		
	**Class 1 *n* (%)**	**Class 2 *n* (%)**	***p*-value^**a**^**	**Class 1 *n* (%)**	**Class 2 *n* (%)**	***p*-value^**a**^**	**Class 1 *n* (%)**	**Class 2 *n* (%)**	***p*-value^**a**^**
**Adversity related to the family of origin**									
Victim of intra-familial sexual violence	7 (18)	41 (16)	0.880	10 (26)	30 (11)	0.027	5 (13)	7 (3)	0.011
Conflicts or tensions with (a) close family member(s)	21 (54)	80 (31)	0.004	24 (62)	105 (40)	0.010	19 (49)	43 (16)	<0.001
Affective distance with (a) close family member(s)	1 (3)	17 (6)	0.486	1 (3)	19 (7)	0.488	2 (5)	9 (3)	0.639
Parental neglect	13 (33)	94 (36)	0.922	15 (38)	81 (31)	0.429	9 (23)	19 (7)	0.004
Inadequate education	9 (23)	48 (18)	0.465	9 (23)	54 (20)	0.706	4 (11)	16 (6)	0.122
Forced to keep or left in ignorance of a secret	2 (5)	20 (8)	0.826	1 (3)	27(1)	0.213	0 (0)	12 (5)	0.376
Separation with (a) close family member(s)	8 (21)	53 (20)	1.000	4 (10)	58 (22)	0.139	3 (8)	25 (9)	1.000
Mental health issues in the family of origin	2 (5)	41 (16)	0.136	2 (5)	42 (16)	0.123	5 (13)	7 (3)	0.011
New partner in parent's life	4 (10)	5 (2)	0.018	1 (3)	2 (1)	0.340	0 (0)	1 (0)	1.000
**Adversity related to affective life**									
End of a romantic relationship	0 (0)	0 (0)	–	3 (8)	8 (3)	0.156	8 (21)	67 (25)	0.647
Tensions and/or arguments in the couple	0 (0)	0 (0)	–	0 (0)	2 (1)	1.000	0 (0)	37 (14)	0.007
Extra-conjugal relationship of the partner	0 (0)	0 (0)	–	0 (0)	1 (0)	1.000	3 (8)	11 (4)	0.402
**Adversity related to procreation and/or siblings**									
Mental health problem in child(ren)	0 (0)	0 (0)	–	0 (0)	0 (0)	–	0 (0)	17 (6)	0.142
Conflicts with child(ren)	0 (0)	0 (0)	–	0 (0)	0 (0)	–	0 (0)	25 (9)	0.055
Change in the frequency of interaction with child(ren)	0 (0)	0 (0)	–	0 (0)	0 (0)	–	2 (5)	12 (5)	0.698
**Personal adversity**									
Victim of psychological violence	7 (18)	62 (23)	0.572	8 (21)	60 (23)	0.917	5 (13)	20 (8)	0.343
Victim of physical violence	3 (8)	27 (10)	0.779	2 (5)	26 (10)	0.553	1 (3)	1 (0)	0.241
Personal physical health problem	5 (13)	44 (17)	0.647	9 (23)	33 (12)	0.084	10 (26)	83 (32)	0.464
Personal psychological health problem	6 (15)	11 (4)	0.441	9 (23)	17 (6)	0.441	6 (15)	3 (1)	0.001
Personal behavioral problems	5 (13)	18 (7)	0.319	17 (44)	45 (17)	<0.001	10 (26)	10 (4)	0.001
Serious accident	0 (0)	5 (2)	1.000	0 (0)	3 (1)	1.000	2 (5)	18 (7)	1.000
Witness or victim of a traumatic event	4 (1)	13 (5)	0.251	4 (1)	17 (6)	0.328	0 (0)	10 (4)	0.371
**Adversity related to academic or professional life**									
Learning disabilities, poor academic performance or school dropout	13 (33)	48 (18)	0.047	17 (44)	72 (27)	0.057	10 (26)	21 (8)	0.004
Relational problems with peers, bullying or harassment at school	3 (8)	22 (18)	0.000	7 (18)	24 (9)	0.094	2 (5)	6 (2)	0.275
Difficulties in finding work	0 (0)	0 (0)	–	0 (0)	0 (0)	–	3 (8)	12 (5)	0.421
Conflicts with colleagues or boss	0 (0)	0 (0)	–	0 (0)	0 (0)	–	0 (0)	11 (5)	0.370
Job loss	0 (0)	0 (0)	–	0 (0)	0 (0)	–	2 (5)	4 (2)	0.173
Unemployment	0 (0)	0 (0)	–	0 (0)	0 (0)	–	6 (15)	53 (2)	0.636
**Adversity related to the extended family**									
Change in the frequency of interaction with a member of the extended family	0 (0)	0 (0)	–	0 (0)	0 (0)	–	6 (15)	32 (12)	0.604
Relational difficulties with a member of the extended family	0 (0)	0 (0)	–	0 (0)	0 (0)	–	2 (5)	34 (13)	0.195
Mental health problems in a member of the extended family	0 (0)	0 (0)	–	0 (0)	0 (0)	–	8 (21)	41 (16)	0.578
Physical health problems in a member of the extended family	0 (0)	0 (0)	–	0 (0)	0 (0)	–	0 (0)	15 (6)	0.284
**Adversity related to social life**									
Difficulty making friends or engaging in social relationships	3 (8)	20 (8)	1.000	7 (18)	26 (10)	0.163	5 (13)	32 (12)	0.799
Loss of (a) friend(s)	1 (3)	5 (2)	0.566	3 (8)	7 (3)	0.125	2 (5)	14 (5)	1.000
Conflictual interactions with (a) friend(s) or rejection	1 (3)	4 (2)	0.500	3 (8)	5 (2)	0.070	9 (24)	28 (11)	0.037
Social isolation	3 (8)	15 (6)	0.713	9 (23)	17 (6)	0.002	7 (18)	73 (28)	0.276
**Adversity related to losses**									
Move or departure	10 (26)	42 (16)	0.132	7 (18)	22 (8)	0.076	9 (24)	34 (13)	0.136
Death of a close relative	1 (3)	6 (2)	1.000	1 (3)	12 (5)	1.000	2 (5)	8 (3)	0.623
Suicide attempt in a relative	1 (3)	5 (2)	0.566	3 (8)	14 (5)	0.467	1 (3)	12 (5)	1.000
Suicide of a relative	8 (21)	19 (7)	0.013	11 (28)	27 (10)	0.004	3 (8)	22 (8)	1.000
**Adversity related to living conditions**									
Precarious living conditions	0 (0)	17 (6)	0.142	0 (0)	14 (5)	0.229	2 (5)	20 (7)	1.000
Homeless	0 (0)	0 (0)	–	0 (0)	1 (0)	1.000	2 (5)	12 (5)	0.698
Financial problems	0 (0)	3 (1)	1.000	0 (0)	4 (2)	1.000	9 (23)	91 (34)	0.219
Legal proceedings against the individual	0 (0)	0 (0)	–	1 (3)	4 (2)	0.500	4 (10)	18 (7)	0.503

a *Proportions are compared with Chi-square or Fisher exact tests depending on the marginal probabilities*.

#### ALE During the Trajectories Break-Point Period

The inter-class comparisons of the ALE that occurred in the 10–14 period is available Table 3, middle columns. The proportion of individuals who experienced social isolation or conflicts within the family unit during early adolescence was significantly higher in class 1 than in class 2 (44 vs. 17%, *p* < 0.001 and 62 vs. 40%, *p* = 0.010, respectively). The two classes also differed significantly in the proportion of members exposed to intra-family sexual violence (26% in class 1 vs. 11% in class 2, *p* = 0.027) or suicide of a relative (28% in class 1 vs. 10% in class 2, *p* = 0.004) between 10 and 14. Finally, we found significantly more behavioral problems in class 1 than in class 2 at these ages (44 vs. 17%, *p* < 0.001).

#### Proximal ALE

With respect to proximal ALE (Table 3, right columns), more class 1 individuals had experienced the following events within the year prior to death, compared with class 2 individuals: conflicts or tensions with a close family member (49% in class 1 vs. 16% in class 2, *p* = 0.001), parental neglect (23% in class 1 vs. 7% in class 2, *p* = 0.004) or conflict with a friend (24% in class 1 vs. 11% in class 2, *p* = 0.037). In addition, there were more frequent behavioral and/or psychological problems in class 1 than in class 2 at the time of death (26 vs. 4% and 15 vs. 1%, respectively, *p* < 0.001). Conversely, a higher proportion of class 2 individuals was exposed to conjugal tensions or arguments in their last year of life (14 vs. 0%, *p* = 0.007).

## Discussion

Relying on a mixed quantitative/qualitative longitudinal approach, we found that adolescence was associated with the bifurcation of two distinct developmental trajectories toward suicide, each corresponding to a specific subpopulation. While both classes had experienced growing adversity during infancy, the divergence of the trajectories increased as of early adolescence. For individuals dying at middle age (class 2), the interplay between adversity and coping strategies stabilized temporarily during young adulthood, possibly due to more efficient allostatic regulation mechanisms. By contrast, suicides in late adolescence (class 1) were preceded by a constant increase in BA, suggesting an acceleration of allostatic alterations.

We see two possible hypotheses to account for the bifurcation that our model predicted at 10–14. First, a substantial proportion of the two classes experienced early ALE that have been identified as strong predictors of suicidal behaviors, including childhood neglect, psychological, physical and sexual violence (28–30). However, individuals who died during late adolescence differed significantly in terms of frequency of conflicts and tensions in the family and arrival of a new partner in one of the parents' lives. Household instability and family stress have been demonstrated to compromise internal safety, emotional regulation or conflict resolution (31), thus altering children's coping abilities (32). Therefore, the divergence of the two trajectories as of puberty may reflect individuals' differential adaptive resources in relation to their early family context. This hypothesis is consistent with repeated evidence that some neuro-biological consequences of early exposure to stress become evident during adolescence, an observation that Lupien et al. coined “incubation/potentiation effect” (14). Alternatively, the steady increase in BA observed in individuals who died in late adolescence could have resulted from some ALE occurring more frequently during the 10–14 period. This would be consistent with the critical vulnerability to adversity that adolescence is known to imply (33–35), due to immature regulation of hormonal responses (36) and greater stress-sensitivity of key brain regions (14, 35).

Interestingly, suicide trajectories were both characterized by the overrepresentation of dependent adverse events [i.e., events that likely occurred non-randomly due to the individual/environment interrelationship (19)], which had a strong social valence. It is thus possible that the social reconfigurations and allostatic maturation that puberty involves have initiated interaction loops between (a) interpersonal dependent ALE (e.g., school difficulties, academic drop out, conflicts with relatives or social isolation) pressurizing adolescents' stress-regulation system and (b) maladaptive coping strategies (reflected by behavioral problems), altering the probability of social ALE to occur (37). Formally speaking, this is equivalent to priming unsteady dynamics in complex open individual–environment systems (38). Mild differences in the early calibration of these systems (39), but also disturbances provoked by ALE disrupting pubertal processes may have resulted in a progressive amplification of the divergence between trajectories. In individuals who died earlier, the cascade (40) may have led to early exhaustion of adaptive mechanisms. Illustrative of such developmental path, Benarous et al. showed that irritability, which can be considered a maladaptive reaction pattern to stress, may contribute to youth suicidal behaviors via three synergic effects (41): it predisposes individuals to suicidal ideations, increases the risk of psychopathology and triggers the transition from suicidal ideation to suicide attempts. In individuals who died later by contrast, the system may have temporarily stabilized, but progressively drifted due to the progressive faltering of allostatic mechanisms, as reflected by the greater proportion of individuals suffering from affective disorders at death. Supporting this interpretation, Neeleman et al. have proposed that the early stages of the suicidal process are mostly driven by environmental influences while the late stages are more autonomous and intricately linked with mental illnesses (42).

Two types of the distal ALE overrepresented in earlier suicide trajectories are worth noting. Almost 25% of individuals who died during late adolescence were victims of intrafamily sexual violence between ages 10 and 14. The literature presents robust evidence that childhood sexual assault leads to higher risk of suicidal outcomes (30). However, our results suggest that the specific effects of sexual victimization during puberty, for which evidence remain scarce, may deserve specific attention. In addition, 20 and 30% of individuals who died in late adolescence were exposed to the suicide of a close relative—including family members—during infancy and young adolescence, respectively. Beyond the genetic and epigenetic endowments and/or shared adverse living conditions that may be implied, such aggregation raises questions about a possible suicidal contagion process. In the past 3 decades, researchers have provided epidemiological (43) and experimental (44) evidence that exposure to a suicide model may contribute to precipitating suicidal behaviors in vulnerable individuals, even several years after the index death (45). In our study, the role of contagion as a proximal risk factor is worth considering, as almost 10% of the whole sample was exposed to the suicide of a close relative in the year prior to their death. However, the fact that the two classes differed in terms of suicide exposure during infancy and adolescence indicates that suicide contagion may also result from a longer term effect, possibly through implicit encryption and retention of the suicide model (46).

To our knowledge, this study is one of the largest psychological autopsy investigations with in-depth collection of ALE that have been carried out so far. However, several limitations need to be considered. First, a common concern about retrospective collection of ALE relates to information or recall biases. In our study, informants may have involuntarily omitted some of the events experienced by their relative due to oblivion or ignorance. The possibility of reporting filters related to stigma, shame, or guilt should also be taken into consideration. Also, the nature of relationship between informants and the deceased persons may have influenced the information that investigators obtained. However, in line with a long tradition of narrative exploration practices, we adopted proven measures to minimize information and/or recall biases and homogenize data collection: systematic semi-structured exploration of pre-specified ALE, minimization of resistances thanks to conversational-style interviews, use of memory anchors, stimulation of the recall efforts with calendars and photos and cross-checking of collected data from various sources (20). Although these precautions may not have fully compensated for the inaccuracies of subjective reporting procedures, it should be noted that possible information biases were likely comparable for the two classes. Of remarkable exception, the proportion of mental health issues in the family of origin as reported for the individuals who died as young adults was oddly low (5% in the 0–14 years period). By contrast, this proportion in the second class (16% in the same period) was more congruent with the literature about the role of parental psychopathology in vulnerability to suicide (47). Rather than a true difference, it is more likely that this observation reflects a differential underreporting effect, possibly in relation to infancy unsteady environments. Family history is indeed especially more difficult to document when children were placed in foster home, separated from siblings, or had little contact with adult family members. The greater frequency of isolation and conflicts in the first class could also have affected the possibility of informants to recall long-term information about parents' mental health and suicidal behaviors. A second limitation is the absence of a comparison group, which prevents us from drawing any conclusion about the role of adolescence in determining whether a person follows a suicidal trajectory. Clearly, this question is of great clinical relevance and would deserve specific developmental investigation, possibly with the same approach as we used here. Finally, the expert rating procedure implied that BA values were available for 5-year periods. This time scale precluded precise examination of the developmental changes that occurred within the adolescence time slot. In future studies, researchers could consider developing alternative methods for transforming ALE data in order to gain in temporal acuity.

Notwithstanding these limits, our results provide original clinically-grounded evidence that developmental transformations characterizing adolescence translate in the suicidal process. These observations highlight the short-, but also longer term relevance that reinforcing clinical attention on adolescents' distress may have in terms of suicide prevention. Targeted intervention could encompass psychotherapeutic support to deal with family dysfunctional interactions and develop more efficient coping strategies.

## Data Availability Statement

The raw data supporting the conclusions of this article will be made available by the authors, without undue reservation.

## Ethics Statement

The studies involving human participants were reviewed and approved by ethics review boards of the Douglas Mental Health University Institute, the Centre Hospitalier Universitaire Sainte-Justine and the Université du Québec en Outaouais. The patients/participants provided their written informed consent to participate in this study.

## Author Contributions

MS was responsible for the original idea of studying life trajectories and conceived the protocol. MS and NC collected the data and organized their quantitative transformation. C-EN conceived the idea of studying the specific role of adolescence in suicide trajectories. C-EN and NC performed preliminary data management. C-EN conducted the analysis with the help of GB. C-EN wrote the manuscript with support from MS, GV, and NC. MS and GV supervised the whole project. All authors contributed to the article and approved the submitted version.

## Conflict of Interest

The authors declare that the research was conducted in the absence of any commercial or financial relationships that could be construed as a potential conflict of interest.

## Abbreviations

ALE: Adverse Life Experiences
BA: Burden of Adversity
SCID-I and SCID-II: Structured Clinical Interview for DSM-IV for Axis I and Axis II disorders, respectively.
