# Supplementary material for: The Role of Adolescence in Development Paths Toward Suicide: Specificities and Shaping of Adversity Trajectories
Source: Front Psychiatry. 2020 Oct 30;11:557131. doi: 10.3389/fpsyt.2020.557131 (PMC7661797; doi:10.3389/fpsyt.2020.557131)
Supplement: Supplementary file 1 [file Table_1.docx]

**Supplementary Table 1.** Estimates of the joint latent class model parameters

| Variables | Levels | Class 1 | | |  | Class 2 | | |
| --- | --- | --- | --- | --- | --- | --- | --- | --- |
|  |  | Mean | *SE* | p value |  | Mean | *SE* | p value |
| **Intercept** |  | 1.25 | 0.30 | < .001 |  | 1.32 | 0.09 | < .001 |
| **Slope 1** |  | 0.51 | 0.09 | < .001 |  | 0.31 | 0.03 | < .001 |
| **Slope 2** |  | 0.72 | 0.21 | .001 |  | -0.08 | 0.06 | .174 |
| **Quadratic 2** |  | -0.03 | 0.05 | .545 |  | 0.07 | 0.02 | < .001 |
| **Hazard mortality ratio** |  | 8.51 | 1.89 | < .001 |  | Ref | - | - |
| **Intercept on** |  |  |  |  |  |  |  |  |
| Gender | Male | 0.00 | 0.21 | > .990 |  | 0.00 | 0.21 | .990 |
|  | Female |  | - | - |  | - | - | - |
| Campaign | Campaign 1 | 0.07 | 0.21 | .730 |  | 0.07 | 0.21 | .730 |
|  | Campaign 2 | 0.69 | 0.23 | .002 |  | 0.69 | 0.23 | .002 |
|  | Campaign 3 | 0.48 | 0.24 | .041 |  | 0.48 | 0.24 | .041 |
|  | Campaign 4 | Ref | - | - |  | - | - | - |
| **Slope on** |  |  |  |  |  |  |  |  |
| Gender | Male | 0.04 | 0.06 | .505 |  | 0.04 | 0.06 | .505 |
|  | Female | Ref | - | - |  | - | - | - |
| Campaign | Campaign 1 | 0.11 | 0.06 | .064 |  | 0.11 | 0.06 | .064 |
|  | Campaign 2 | 0.07 | 0.07 | .266 |  | 0.07 | 0.07 | .266 |
|  | Campaign 3 | 0.48 | 0.08 | < .001 |  | 0.48 | 0.08 | < .001 |
|  | Campaign 4 | Ref | - | - |  | - | - | - |
| **Quadratic term on** |  |  |  |  |  |  |  |  |
| Gender | Male | 0.01 | 0.02 | .461 |  | 0.01 | 0.02 | .461 |
|  | Female | Ref | - | - |  | - | - | - |
| Campaign | Campaign 1 | 0.03 | 0.02 | .126 |  | 0.03 | 0.02 | .126 |
|  | Campaign 2 | 0.04 | 0.02 | .034 |  | 0.04 | 0.02 | .034 |
|  | Campaign 3 | 0.07 | 0.06 | .275 |  | 0.07 | 0.06 | .275 |
|  | Campaign 4 | Ref | - | - |  | - | - | - |
| **Hazard on** |  |  |  |  |  |  |  |  |
| Gender | Male | 1.51 | 0.83 | .067 |  | 1.51 | 0.83 | .067 |
|  | Female | Ref | - | - |  | - | - | - |
| Campaign | Campaign 1 | 0.25 | 1.28 | .843 |  | 0.25 | 1.28 | .843 |
|  | Campaign 2 | 2.93 | 1.78 | .101 |  | 2.93 | 1.78 | .101 |
|  | Campaign 3 | 9.83 | 1.67 | < .001 |  | 9.83 | 1.67 | < .001 |
|  | Campaign 4 | Ref | - | - |  | - | - | - |

*Note.* *SE* = Standard Error, Quadratic 1 term is omitted as not specified in the model.
